# Adjudication of exacerbations in bronchiectasis using modified EMBARC criteria in the CLEAR Trial

**DOI:** 10.1183/23120541.00361-2026

**Published:** 2026-09-28

**Authors:** Brenda O'Neill, Rohan Anand, Jeremy S. Brown, Frances Butler, Lorcan McGarvey, Cecilia M. O'Kane, Mike Clarke, Danny F. McAuley, Adam T. Hill, Anthony De Soyza, James D. Chalmers, Andrew Jackson, Ben McCullough, Fiona Copeland, J. Stuart Elborn, Judy M. Bradley, on behalf of the CLEAR Investigator team

**Affiliations:** 1 Institute of Nursing and Health Research, Ulster University, Belfast, UK; 2 Wellcome-Wolfson Institute for Experimental Medicine, Queen's University Belfast, Belfast, UK; 3 UCL Respiratory, University College London, London, UK; 4 York Teaching Hospital NHS Foundation Trust, York, UK; 5 Centre for Public Health, Queen's University Belfast, Belfast, UK; 6 Northern Ireland Clinical Trials Unit, Belfast, UK; 7 Centre for Inflammation Research, University of Edinburgh, Edinburgh, UK; 8 Freeman Hospital Newcastle, Newcastle, UK; 9 Radcliffe Department of Medicine, University of Oxford, Oxford, UK; 10 School of Medicine, University of Dundee, Dundee, UK; 11 Wellcome Trust-Wolfson Northern Ireland Clinical Research Facility, Queen's University Belfast, Belfast, UK; 12 PCD Family Support Group, Ciliopathy Alliance, London, UK; 13 Joint senior authors; 14 For a list of the CLEAR Investigator team members and their affiliations see the supplementary material

## Abstract

**Introduction:**

The EMBARC consensus criteria can be used to identify and categorise pulmonary exacerbations (PEs) in clinical trials. This paper describes methods used to independently adjudicate PEs using modified EMBARC criteria in a large multicentre bronchiectasis trial (CLEAR ISRCTN89040295).

**Methods:**

The Respiratory and Systemic Symptoms Questionnaire was used to objectively collect signs and symptoms at the onset of a PE. A mEMBARC PE was defined as ≥3 symptoms, with symptoms present for ≥48 h and prescription of an antibiotic. Individual sites recorded antibiotic use for PEs. An adjudication panel independently adjudicated and then agreed on PEs according to mEMBARC criteria. Data required to determine PEs using modified Fuchs criteria was also collected.

**Results:**

Sites reported 462 PEs from 203 patients reported in the CLEAR trial. The adjudication panel categorised these exacerbations as: 239 out of 462 (52%) fully qualifying mEMBARC PEs (meeting all mEMBARC criteria), 32 out of 462 (7%) partially qualifying mEMBARC PEs (≤3 symptoms present for ≥48 h or ≥3 symptoms present for <48 h and antibiotics prescribed) and 191 out of 462 (41%) non-mEMBARC PEs (antibiotics prescribed but symptoms and duration not met). When data were classified using modified Fuchs criteria a sensitivity analysis produced results that were consistent with the main trial results.

**Conclusion:**

PEs are increasingly used as the primary outcome in bronchiectasis trials, making accuracy in their identification essential. More rigorous capture of exacerbation symptoms and implementation of a standardised and reproducible decision-making process, supported by an independent adjudication panel, is important for capturing PEs and ensuring the integrity of trial outcomes.

## Shareable abstract

**EMBARC criteria along with an independent adjudication panel substantially changes the total number of PEs captured in a bronchiectasis trial. Standardised processes and electronic data capture could enhance the validity of reporting PEs in trials.** https://bit.ly/4fgdWTT

## Introduction

Pulmonary exacerbations (PEs) have been used extensively as an outcome in bronchiectasis-related research, with recent large phase 3 clinical trials using PEs as a primary outcome [1, 2]. PEs also rank highly within existing core outcome sets for bronchiectasis clinical trials [3]. However, there is ambiguity surrounding the definition and specific criteria for recognising and categorising PEs in clinical trials. The definition varies between organisations and clinicians with regards to what signs, symptoms and measurements contribute towards a diagnosis of an exacerbation [4, 5].

In clinical practice guidelines there is inconsistency in how PEs are defined [6–9]. In clinical trials, definitions were historically modelled on Fuchs criteria, which originated in a trial of rhDNAse in cystic fibrosis [10] with O'Donnell and colleagues [11] modifying the Fuchs criteria for use in bronchiectasis; others define PEs purely in terms of the decision to treat with antibiotics [12]. In 2017 a consensus definition was developed for measuring PEs specifically for adult bronchiectasis trials [13] with the purpose of standardising PEs as a clinical research outcome. These criteria, termed the European Multicentre Bronchiectasis Audit and Research Collaboration (EMBARC) international consensus criteria, are summarised in table 1. Trials (n=14) published since 2017 have used PEs as the primary outcome, with five out of 14 trials using the EMBARC criteria; although it is noted that some published trials had enrolment periods before the criteria were published (supplementary material) [2, 14–17]. Importantly the EMBARC definition has been already adopted by trialists [2, 14–17], and regulators have guided expectations for clinical studies specific to the identification of exacerbations despite relatively few efforts to validate/explore the performance of definitions such as the EMBARC definition [18]. The first licensed therapy in bronchiectasis has recently been achieved, and it used the EMBARC criteria to define PE [19].

**TABLE 1:** Classification of pulmonary exacerbation (PE) categories

Classification of pulmonary exacerbation	Criteria for PE category
**Full mEMBARC PE**	Deterioration in at least three symptoms **AND** duration of symptoms is for 48 h or more **AND** antibiotics
**Partial mEMBARC PE**	Deterioration in at least three symptoms Duration of all the reported symptoms is for 48 h or more **AND** antibiotics **OR** duration of all the reported symptoms is for 48 h or more **AND** antibiotics^#^
**Non-mEMBARC PE**	Reported as an exacerbation and antibiotics were prescribed, but full or partial criteria not met
**Unreported/missed PEs**	PEs recorded on the concomitant medications log when the patient reported taking antibiotics during a site visit appointment but they had previously not contacted the site to inform them or enable categorising the exacerbation
**No PE**	Information on trial database but no antibiotics were prescribed

^#^: *i.e.* if antibiotics were prescribed and there were either one or two symptoms, AND these reported symptoms have been present for >48 h this is a partial exacerbation.

The variation in PE definitions makes it difficult to compare, contrast and combine the findings for PEs across different studies [20]. This limits the use of meta-analyses to resolve important uncertainties about treatment effects and determine what treatments work. Trials outside of bronchiectasis have detailed the use of End-point Adjudication Committees or Algorithms in the context of decision making about the primary end-point [21–23]. These processes help to ensure consistency and independence when agreeing and reporting trial outcomes and can help optimise the integrity of the trials by providing standardised adjudication of outcomes that have been shown to differ from data collected from assessment by site investigators alone [21, 23]. In bronchiectasis, three out of 14 trials that used PEs as the primary outcome [1, 2, 24] used some form of adjudication for their exacerbations (supplementary material). However, only one of 14 trials [2] used both the EMBARC criteria as well as adjudication of their exacerbations.

In this paper we aimed to describe the methods used to implement the EMBARC criteria in a clinical trial, including the use of an independent adjudication panel in a large multicentre bronchiectasis trial (CLEAR, ISRCTN89040295). This information will assist clinicians and researchers to consider the application of a similar process, including an adjudication panel, to consistently capture the EMBARC criteria for PEs in future trials. One modification (in consultation with the lead author of the definition [13]) was to limit the “change in treatment” specifically to the initiation of antibiotics only, and therefore we refer to modified EMBARC (mEMBARC) throughout the manuscript.

Our objectives were to:

1) Describe the methods used to collect PEs from sites and categorise these according to mEMBARC criteria as full, partial or non-mEMBARC PE (table 1).
2) Describe the use of an independent adjudication panel and any differences in the numbers of PEs that meet the full mEMBARC criteria adjudicated by the panel compared to the total number of trial PEs.
3) Evaluate the impact of PE categorisation on the CLEAR trial outcome when using the traditional modified Fuchs criteria compared with the adjudicated mEMBARC method.

## Methods

This analysis was completed using data from the CLEAR clinical trial. CLEAR is a large multisite, phase 3 trial exploring mucoactives for bronchiectasis at 20 sites in the UK [25, 26]. The primary outcome was the number of mEMBARC-defined fully qualifying PEs over 1 year following enrolment into the trial.

Sites had agreed to provide standard care per the British Thoracic Society (BTS) guidelines [13], and all patients were expected to have been taught an airway clearance technique and provided with an airway clearance plan (with usual symptoms noted), and if a patient did not have a regular airway clearance regimen, they were taught active cycle of breathing techniques. Patient partners were involved in the design, reporting and dissemination plans of our research.

### Data collection for PEs

At baseline and at each subsequent study visit patients completed the Respiratory and Systemic Symptoms Questionnaire (RSSQ) [27] to capture signs and symptoms during stability and during PEs. All participants were familiar with their usual symptoms and given a study card with details to contact their study team anytime they feel that they are experiencing symptoms of a potential exacerbation and/or before commencing any antibiotics. If this occurred, then an additional RSSQ was completed and used to objectively collect relevant signs and symptoms including symptom duration required for the mEMBARC criteria (table 2). Sites were advised that if the patients’ symptoms were <48 h they should follow-up again at 48 h. The RSSQ questionnaire was completed remotely with the study team over the telephone or on site (unscheduled visit).

**TABLE 2:** Summary of the three parts of the mEMBARC definition for bronchiectasis pulmonary exacerbations (PEs)

Criteria for the mEMBARC definition^#^	
**Part 1: Symptoms**	Patient must present with deterioration in three or more of the six following symptoms: 1) Cough 2) Sputum volume and/or consistency 3) Sputum purulence 4) Breathlessness and/or exercise tolerance 5) Fatigue and/or malaise 6) Haemoptysis
**Part 2: Time**	Symptoms must be present for at least 48 h
**Part 3: Treatment^#^**	Prescription of antibiotics

The EMBARC definition consists of three parts which must all be satisfied for a fully qualifying exacerbation. For separate exacerbations there must be an unequivocal resolution of symptoms from the first event and >14 days to the commencement of a subsequent event. If this criterion is not met, then the exacerbation is counted as a single continuous event ^#^: one modification (in consultation with the lead author of the definition [13]) was to limit the “change in treatment” (Part 3) specifically to the initiation of antibiotics only.

The site staff were to use the signs and symptoms information collected from the RSSQ results to assess for an exacerbation against the mEMBARC criteria (table 3) and discuss these findings and the status of the patient with physicians at the site to direct any prescription for antibiotics or whether any other change to the patient's treatment is required and communicate this to the patient.

**TABLE 3:** EMBARC scoring system [13] linked to the Respiratory and Systemic Symptoms Questionnaire (RSSQ) [21]^#^

Domain	Question number (from RSSQ)	Answers that qualify as deterioration (from RSSQ)	Is time >48 h? (Yes or No)	Deterioration in domain? (Yes or No)
**Cough**	5.1 and/or 5.2	“much harder” or “a little harder” or “this is a new symptom”	□	□
		“much more often” or “a little more often” or “this is a new symptom”		
**Sputum volume and/or consistency**	1 and/or 2.1	“much more” or “a little more” or “this is a new symptom”	□	□
		“much thicker” or “a little thicker” or “this is a new symptom”		
**Sputum purulence**	2.2	“worse” or “this is a new symptom”	□	□
**Breathlessness and/or exercise tolerance**	7 and/or 6	“much more difficult” or “a little more difficult”	□	□
		“much harder” or “a little harder”		
**Fatigue and/or malaise**	8	“much more tired” or “a little more tired”	□	□
**Haemoptysis**	4	“large increase” or “a little increase” or “this is a new symptom”	□	□
			Total deteriorations	/6

If more than one question was listed with “and/or” as a separator, then only one of the questions and respective answers qualified as a deterioration in that domain. ^#:^ note the linkage to RSSQ questions may not align perfectly to how EMBARC was designed to be used.

Patients mostly had a course of antibiotics at home to facilitate management of PEs remotely. During the trial, the individual site recorded all antibiotics use in the concomitant medication log. The end of the PE was defined as the time when the prescribed antibiotic course was completed. At this time another RSSQ questionnaire (end exacerbation RSSQ) was completed with the patient to determine resolution of the PE. If further antibiotics were prescribed (within 14 days of end first course), the PE would be regarded as a continuous single event. An overview of PE data collection is given in figure 1.

**FIGURE 1:**
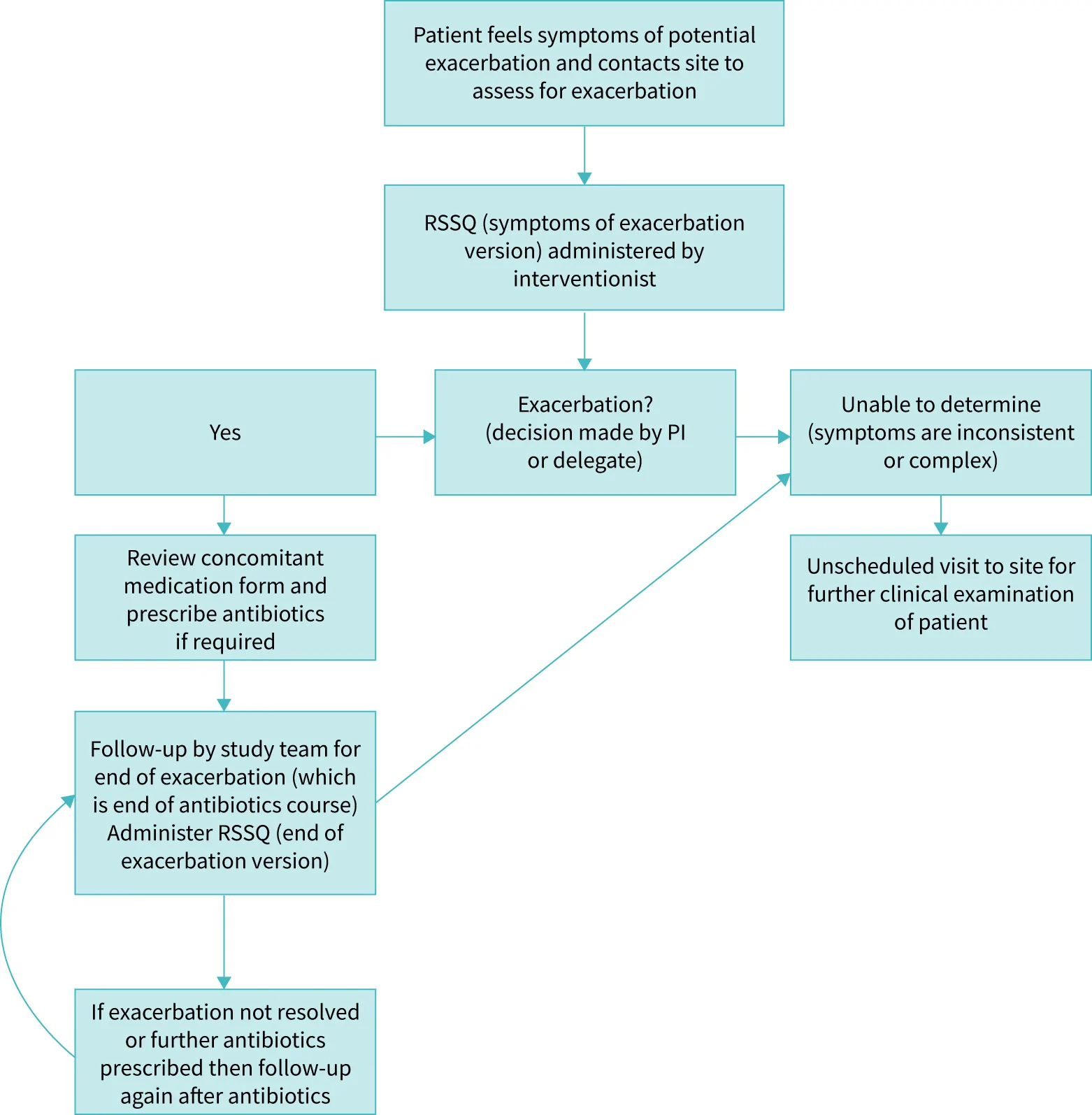
Schematic diagram of remote data collection for pulmonary exacerbations (PEs) during the CLEAR trial. RSSQ: Respiratory and Systemic Symptoms Questionnaire; PI: principal investigator.

Data collection enabled classification of PE exacerbations aligned to both the mEMBARC criteria and modified Fuchs criteria (table 2, table 4). The modified Fuchs criteria were not adjudicated and were defined by the presence of abnormalities in at least four of seven domains. Domain 7 was defined as a ≥10% reduction in forced expiratory volume in 1 s (FEV_1_) relative to a previously recorded spirometry value (table 4). Research staff asked the patient to undertake a lung function test using their home spirometer (*my*SpiroSense; PARI Pharma), and these data were used to determine the percentage change of FEV_1_ over time.

**TABLE 4:** Modified Fuchs scoring system

Domain	Question number(s) (from RSSQ)	Answers that qualify as deterioration (from RSSQ)	Is time >48 h (Yes or No)	Deterioration in domain? (Yes or No)
**Change in sputum production (consistency, colour, volume or haemoptysis)**	2.1 and/or 2.2 and/or 1 and/or 4	“much thicker” or “a little thicker”	□	□
		“worse”		
		“much more” or “ a little more”		
		“large increase” or “a little increase”		
**Increased dyspnoea (chest congestion or shortness of breath)**	3 and/or 7	“large increase” or “little increase”	□	□
		“much more difficult” or “a little more difficult”		
**Increased cough**	5.1 and/or 5.2	“much harder” or “a little harder”	□	□
		“much more often” or “a little more often”		
**Fever (>38°C)**	9	“yes”	□	□
**Increased wheezing**	15 (additional question)	“large increase” or “little increase”	□	□
**Decreased exercise tolerance, malaise, fatigue or lethargy**	6 and/or 8	“much harder” or “a little harder”	□	□
		“much more tired” or “a little more tired”		
**FEV_1_ decreased 10% from a previously recorded value^#^**	MySpiroSense Spirometer	“yes”	N/A	
			Total deteriorations	/7

A modified Fuchs exacerbation was classified as deterioration in at least four of the seven domains. RSSQ: Respiratory and Systemic Symptoms Questionnaire; FEV_1_: forced expiratory volume in 1 s. ^#^: domain 7 was defined as a ≥10% reduction in FEV_1_ relative to a previously recorded spirometry value, calculated as a previously recorded value from a last stable study visit where possible, as these are the main lung functions we will know are done satisfactorily. If a study visit is not considered stable then nearest stable lung function to visit will be considered [4, 28]. The percentage decrease will be calculated as: $\frac{\;\;\mathrm{FE}V_{1}\;\;\mathrm{previously}\;\mathrm{recorded}\text{–}\mathrm{FE}V_{1}\;\mathrm{at}\;\mathrm{start}\;\mathrm{of}\;\mathrm{exacerbation}\;}{\mathrm{FE}V_{1}\;\mathrm{previously}\;\mathrm{recorded}}\times 10$

### The adjudication panel

The purpose of the panel was to independently adjudicate PEs aligned to the mEMBARC criteria. The mEMBARC criteria were applied within the trial as per the original definition (table 2). In brief the criteria have three parts: Part 1 Symptoms (≥3 symptoms), Part 2 Time (symptoms present for 48 h), Part 3 Treatment (use of antibiotic). The adjudication of exacerbation-related events in CLEAR took place retrospectively. The decision-making panel members included two consultant respiratory physicians and one specialist respiratory physiotherapist (J. Brown, F. Butler throughout the trial; L. McGarvey first half, C.M. O'Kane second half of trial), all with extensive experience in the treatment and management of chronic respiratory disease and independent of the design or involvement of the CLEAR trial. Training was provided prior to their first adjudication meeting which included familiarising members with the CLEAR trial, the exacerbation management plan and the panel operating procedures and terms of reference. It included examples of how potential events could be presented to the panel.

The first adjudication meeting was planned within the first 6 to 12 months with regular meetings thereafter (usually every 3 months or when a batch of data were ready) until the study ended. The panel members independently reviewed each classification sheet to score each PE as either: “fully qualifying mEMBARC exacerbation”, “partially qualifying mEMBARC exacerbation”, “non-mEMBARC exacerbation” or “No exacerbation” (table 1). Individual panel member decisions about each potential PE were summarised to record whether there was majority agreement (two out of three on panel), unanimous agreement (three out of three on panel), or no agreement about the category of PE exacerbation. At each meeting the panel aimed to discuss and reach consensus about the presence/absence of an exacerbation. The consensus discussion involved reviewing the exacerbation category that each individual panel member provided, identifying any individual errors or mistakes, reconsidering the data and discussing each criteria one at a time, reflecting on similar past presentations of exacerbation data, discussing any ambiguities and finally reaching consensus.

### Data processing and analysis of exacerbation events

Prior to each adjudication panel meeting the relevant PE and RSSQ data were provided by the Clinical Trials Unit. The responses from the RSSQ matched to the relevant mEMBARC criteria (Part 1 and Part 2) and the information was prepared for bespoke data collection “classification sheets” for the panel members to review. This included any clinical notes that sites had recorded as part of the PE event.

Descriptive statistics summarised the results. The number of PEs that met the full mEMBARC criteria adjudicated by the panel were compared to the total number of PEs identified from the site. The number of PEs that were counted as a single continuous event was recorded.

For the number of exacerbations defined using the Fuchs criteria a *post hoc* analysis was repeated with modified intention-to-treat (ITT) as per the CLEAR trial [26] and groups were compared over the 52 weeks using Negative Binomial Regression: 1) participants who received hypertonic saline compared to those who did not; and 2) participants who received carbocisteine compared to those who did not.

## Results

There were 462 PEs from 203 patients reported in the clinical record form. Adjudicated PE results were 239 out of 462 (52%) mEMBARC PEs (fully qualifying mEMBARC exacerbation), 32 out of 462 (7%) partially qualifying mEMBARC PEs (≤3 symptoms or symptoms present for <48 h and antibiotics prescribed) and 191 out of 462 (41%) non-mEMBARC PEs (antibiotics prescribed but symptoms and duration not met). A proportion of the non-mEMBARC PEs (111 out of 462; 24%) occurred when patients had taken antibiotics (per medication logs) but had not notified the site to provide details about symptoms (supplementary table S1). 10 PEs were adjudicated and counted as a single continuous event (*i.e.* antibiotic prescribed within 14 days of previous antibiotic, table 1) and, therefore, not included in the total PEs.

Each adjudication panel member (n=3) independently classified the PEs as fully qualifying mEMBARC, or partially qualifying mEMBARC or non-mEMBARC. There was excellent agreement amongst the three raters giving confidence in our panel results (weighted kappa 0.947 with 95% confidence interval 0.927–0.966). When the individual data were summarised, there were 98% unanimous or majority panel decisions (309 out of 462; 84% unanimous and 63 out of 462; 14% majority decisions) and nine out of 462 (2%) where each of the three panel members had made different classifications; reasons for these different individual decisions included, *e.g.* recording error, ambiguity about the number and duration of symptoms and a panel member not making a decision until the opportunity for discussion at the meeting. The panel met to discuss and reach consensus about the final PE classification for each participant for all PEs.

There were 34 occasions where sites recorded data or entered a comment into the part of the trial database that collects PE data, although no antibiotics were provided; these were checked as part of quality monitoring and recorded as “no PE” for completeness.

Classification according to the Fuchs criteria was undertaken where sufficient RSSQ data were available (table 4). Of 234 fully qualifying mEMBARC exacerbations with RSSQ data available to enable Fuchs classification, 224 (95.7%) were also classified as modified Fuchs exacerbations. Of 27 partially qualifying mEMBARC exacerbations with RSSQ data available, 20 were also classified as Fuchs exacerbations (74.1%). The CLEAR trial found no statistically significant effect of either hypertonic saline or carbocisteine on reducing exacerbation frequency using fully qualifying mEMBARC, and sensitivity analyses using different categories of mEMBARC exacerbation definitions gave consistent results [26]. *Post hoc* analyses using Fuchs-defined PEs were aligned with those based on mEMBARC criteria.

## Discussion

The number of PEs per year and/or time between PEs are important outcomes in bronchiectasis and are linked to higher symptom burden, poorer quality of life and higher morality [29, 30]. This study showed that the use of both the mEMBARC criteria and an adjudication panel can dramatically influence the total number of PEs in a clinical trial in bronchiectasis. Just over half (52%) of the trial PEs were adjudicated as full mEMBARC PEs, and a proportion of PEs (24%) were classified as non-mEMBARC exacerbations when patients had taken antibiotics but details about symptoms were not available to match against the mEMBARC criteria [13]. When data were classified using different categories for PEs (fully qualifying, partially qualifying and non-mEMBARC PEs) or using different criteria (modified Fuchs) the sensitivity analysis produced results that were consistent with the main trial results [26].

The results support the need for robust processes to identify and record PEs in trials in bronchiectasis, and the value of independent adjudication of mEMBARC PEs in clinical trials in bronchiectasis. Implementing the mEMBARC criteria with an adjudication process could lead to a more standardised approach to defining PE in future trials. Our process has been fully detailed to provide a useful framework which other researchers could consider and easily adopt for implementing the consensus-based mEMBARC criteria for PEs in trials.

Our use of a blinded adjudication panel aligns with practices in other high-quality trials to strengthen identification of important clinical end-points, minimise bias and enable better accuracy for comparison across trials [1, 2, 21–24, 31]. Importantly the reliance on patient-reported outcomes as the primary outcome in a CTIMP trial is challenging, and the implementation of adjudication panels offers regulators more confidence in validity and robustness of data. The implementation of adjudication panels requires planning and time from a dedicated independent panel; however, the improvement in credibility of trials that use this approach makes the investment worthwhile and strategically important in future research.

The high number of PEs treated with antibiotics despite not meeting the full mEMBARC criteria or where data on symptoms were not available for classification is challenging. Despite outlining a clear process, *a priori*, patients sometimes still took antibiotics without notifying trial sites (unreported/missed exacerbations which were classified as non-mEMBARC) and so there were no data about symptoms on these occasions; importantly we still managed to capture this antibiotic use on our medications log. Chalmers and colleagues [2] have recently used patient-completed electronic diaries to record daily symptoms with embedded computer programming to detect an increase in symptoms alerting to a possible PE [2], and this could further improve the accuracy of the application of the mEMBARC criteria. If rigorous data capture processes are not embedded in future trials, there could be a risk of under- or over-reporting PEs, and this could mean that a new medication or therapy is deemed effective when it actually may not be and vice versa. Decentralisation of future trials will represent an important consideration for bronchiectasis trials to improve their logistics and efficiency [32]. Implementation of tools like the Bronchiectasis Exacerbation and Symptoms Tool (BEST) diary could help to minimise or avoid missing symptoms data [33] and remote resources could facilitate data collection such as use of telephones, mobile applications or websites to collect data [2, 34].

The adjudication panel further classified PEs as partial mEMBARC (7%) or non-mEMBARC PEs (17%), and while we are unsure of the value of these specific categories, their inclusion enabled the panel to highlight the range of presentations of PEs. Our statistical analysis was based on the full mEMBARC criteria for PEs for the primary analysis, but a sensitivity analysis compared trial outcomes between full mEMBARC-adjudicated PEs and all other PE classifications with antibiotic use and the results were similar. We also found that when we used the Fuchs definition for PEs in our analysis the trial outcome was the same (*i.e.* no statistically significant difference between groups as was shown in the mEMBARC analysis [26]) giving confidence that spirometry (included in Fuchs but not mEMBARC criteria) does not appear to be an essential criterion in the assessment of PEs.

### Strengths

We captured data to ensure we could distinguish between antibiotics taken for PEs as opposed to antibiotics for another reason to minimise the impact on the trial results. We also included opportunities to optimise data collection such as remote monitoring and remote data collection, *e.g.* collection of symptoms (RSSQ) by phone reducing the need for face-to-face study visits. Regular staff training and updates were provided for research staff and principal investigators (PIs) along with the provision of a trial exacerbation management guide [25]. If a local PI was not available when participants contacted sites about a potential PE, the site could contact the chief investigator to support consistency in decision making about PEs.

### Limitations

There are some limitations that warrant further discussion. First, we relied on patients to tell us about any change in symptoms prior to starting antibiotics and/or to tell us if they have already taken antibiotics. To support this, research staff reminded patients at each appointment about their usual symptoms and to contact the site about any change in symptoms. A specific exacerbation queries log was retained so that any anomalies in data reports for an individual participant or site could be checked. Despite this we missed obtaining data about symptoms if patients took antibiotics but did not contact us and if so, we could only adjudicate these as non-mEMBARC exacerbations. The inclusion of a patient diary to capture symptoms could have avoided this. Also, future use of electronic records shared across primary and secondary care would optimise accurate data about prescription of antibiotics instead of relying on patient recall. Facilitation of patient-held antibiotics during a trial may also warrant further debate.

Second, there was the potential that some PEs categorised as partial mEMBARC or non-mEMBARC were in fact full PEs perhaps if, for example, the patient did not report taking an antibiotic, or the patient did not call the site within 48 h, or they could not accurately recall when symptoms started, or they felt too unwell. This could reflect a limitation related to the validity of the mEMBARC criteria [13]. Therefore, it is important that our analysis for the CLEAR trial included a sensitivity analysis to combine all categories of PEs that were treated with an antibiotic. Third, the adjudication process was retrospective and there was no opportunity by the adjudication panel to contact participants to verify or obtain additional information which might be an option in real time. Despite these limitations, our information may help other clinicians and researchers to consider the application of a similar adjudication process to robustly standardise the implementation of the consensus-based mEMBARC criteria in future trials.

### Conclusion

PEs are increasingly used as the primary outcome in bronchiectasis trials, making accuracy in their identification essential. More rigorous capture of exacerbations symptoms and implementation of a standardised and reproducible decision-making process, supported by an independent adjudication panel, is important for capturing PEs and ensuring the integrity of trial outcomes.

## Supplementary material

**Please note:** supplementary material is not edited by the Editorial Office, and is uploaded as it has been supplied by the author. CLEAR investigator team 00361-2026.SUPPLEMENT Supplementary material 00361-2026.SUPPLEMENT2 Table S1 00361-2026.SUPPLEMENT3
