# Supplementary material for: Adjudication of exacerbations in bronchiectasis using modified EMBARC criteria in the CLEAR Trial
Source: ERJ Open Res. 2026 Sep 28;12(5):00361-2026. doi: 10.1183/23120541.00361-2026 (PMC13617598; doi:10.1183/23120541.00361-2026)
Supplement: Supplementary file 1 [file 00361-2026.SUPPLEMENT.pdf]

CLEAR INVESTIGATOR TEAM

Damian G. Downey, Belfast City Hospital, Belfast Health and Social Care Trust

Rory Convery, Southern Health and Social Care Trust

James Chalmers, Ninewells Hospital and Medical School

Martin Kelly, Western Health and Social Care Trust

Terence McManus, Western Health and Social Care Trust

John R. Hurst, Royal Free London NHS Foundation Trust

Anthony De Soyza, Freeman Hospital, Newcastle upon Tyne

Adam T. Hill, Royal Infirmary Edinburgh

Michael R. Loebinger, Royal Brompton Hospital

Muhammad Anwar, Princess Alexandra Hospital

Mary Carroll, Southampton General Hospital

Timothy Gatheral, Royal Lancaster Infirmary

Anita L. Sullivan, Queen Elizabeth Hospital, Birmingham

William Flight, Churchill Hospital, Oxford

Nick P. Talbot, Churchill Hospital, Oxford

Mitra Shahidi, Stoke Mandeville Hospital

Andrea Alina Ionescu, Royal Gwent Hospital, Newport

Jamie Duckers, University Hospital Llandough, Cardiff & Vale

Mohamed Etumi, Blackpool Victoria, Blackpool Teaching Hospitals

John Steer, Northumbria Healthcare NHS Foundation Trust

Veeresh Patil, Milton Keynes University Hospital

Milan Bhattacharya, Milton Keynes University Hospital
