# Supplementary material for: Adjudication of exacerbations in bronchiectasis using modified EMBARC criteria in the CLEAR Trial
Source: ERJ Open Res. 2026 Sep 28;12(5):00361-2026. doi: 10.1183/23120541.00361-2026 (PMC13617598; doi:10.1183/23120541.00361-2026)
Supplement: Supplementary file 2 [file 00361-2026.SUPPLEMENT2.pdf]

Title: Adjudication of pulmonary exacerbations in bronchiectasis using the EMBARC consensus exacerbation criteria: data from the CLEAR Trial

Brenda O'Neill[1], Rohan Anand[2], Jeremy S Brown[3], Frances Butler[4], Lorcan McGarvey[5], Cecilia M O'Kane[2], Mike Clarke[5,6], Danny F McAuley[2], Adam T Hill[7], Anthony De Soyza[8], James D Chalmers[9,10], Andrew Jackson[6], Ben McCullough[11], Fiona Copeland[12], J Stuart Elborn[2*], Judy M Bradley [2,12, ^, *], on behalf of the CLEAR trial Investigators

1. Institute of Nursing and Health Research, Ulster University, Belfast, UK
2. Wellcome-Wolfson Institute for Experimental Medicine, Queen's University Belfast, UK
3. UCL Respiratory, University College London, UK
4. York Teaching Hospital NHS Foundation Trust, UK
5. Centre for Public Health, Queen's University Belfast, UK
6. Northern Ireland Clinical Trials Unit, Belfast, UK
7. Centre for Inflammation Research, University of Edinburgh, UK
8. Freeman Hospital Newcastle, Newcastle, UK
9. Radcliffe Department of Medicine, University of Oxford, United Kingdom
10. School of Medicine, University of Dundee, Dundee, UK
11. Wellcome Trust-Wolfson Northern Ireland Clinical Research Facility, Queen's University Belfast, UK
12. PCD Family Support Group, Ciliopathy Alliance, London, UK

Supplement 1
Methods used to capture pulmonary exacerbations when used as a primary OM in clinical trials in Bronchiectasis since 2017.

Search methods to find trials:

Scoping search on PubMed for clinical trials in BE since 2017 that used exacerbations as part of the primary outcome measure. Reference lists of retrieved papers were searched. In addition, clinicaltrial.gov was searched up to November 2025.

Search string for PubMed (adjusted as appropriate for clinicaltrials.gov):

Date: 25th November 2025.

("bronchiectasis"[MeSH] OR "bronchiectasis"[Title/Abstract])

AND ("exacerbation"[Title/Abstract] OR "exacerbations"[Title/Abstract])

AND ("clinical trial"[Publication Type] OR "randomized controlled trial"[Publication Type])

AND ("2017/01/01"[Date - Publication] : "3000"[Date - Publication])

AND ("primary outcome"[Title/Abstract] OR "primary endpoint"[Title/Abstract])

Results:

PubMed records returned: 21. Clinicaltrials.gov records returned: 19.

Only those presented below in the table below had exacerbations as the primary outcome.

To our knowledge, 14 trial reports published since 2017 have used PEs as the primary outcome, with 5 using the EMABRC criteria (Supplemental Table 1). 1 report we located since 2017 that used EMABRC as a primary outcome used an adjudication panel (ASPEN) and 2 trials that did not use EMABRC criteria did use a form of adjudication for their exacerbations. There were 5 ongoing clinical trials found on clinicaltrials.gov, 2 of which are listed as using EMBARC criteria. It is important to note that some published trials had enrolment periods before the criteria was published.

Supplement Table 1 Results: Pulmonary exacerbations when used as a primary OM in clinical trials in Bronchiectasis since 2017

Trial name and intervention	Patient population	Method for defining a Pulmonary Exacerbation	Used an adjudication panel?	Used EMBARC criteria?
**Completed trials**				
PROMIS-I and PROMIS-II [1]<br><br>(Inhaled colistimethate sodium for Pseudomonas)	Adult BE with P. aeruginosa and ≥2 exacerbations requiring oral antibiotics or 1 requiring IV antibiotics in the previous year.	Presence of at least 3 of the following for 24 hours: **increased cough**, **sputum volume/consistency/purulence**, haemoptysis, **wheezing**, **dyspnoea**, **fatigue/malaise**, fever (≥38°C).	Yes. Blinded Data Assessment Committee (DAC) reviewed reported PEs, & proposed data queries for investigators to respond to. Determination whether or not a PE met the protocol definition remained with site Principal Investigator	No
BF-1T26 [2] (NCT03643302)<br><br>(Bronchoscopic airway clearance)	Adult BE as per adult bronchiectasis guidelines, with a history of acute exacerbation, eligible for bronchoscopy, and willing to undergo B-ACT therapy.	At least 3 of the following for **48 hours**: **Cough**, **sputum changes**, **breathlessness**, **fatigue**, **haemoptysis**, and clinician determination to alter treatment.	No	Yes
Target-ABC [3,4] (NCT03262142)<br><br>(Investigated systemic antibiotics for Pseudomonas)	Adults with COPD, non-CF bronchiectasis, or asthma and current P. aeruginosa-positive lower respiratory culture.	Time to Exacerbation: Time from day 20 to 365 post-randomisation to prednisolone/antibiotic use or death.	No	No
Nicolini[5]	Adults (18 to 80 years) with BE confirmed by chest CT	Deterioration of at least three respiratory symptoms (**cough, increased sputum**	No	No

(High frequency chest wall oscillation devices)		**production/volume/purulence/change in viscosity,** with or without increasing wheeze, **increased dyspnea, hemoptysis**, and chest pain) for >24 hours and/or systemic complaints, such as fever, and alterations in chest radiograph.		
BEST-2 [6]<br><br>(Amoxicillin-clavulanate versus azithromycin)	Children (aged 1–19 years) Children (1-19 years) with clinical bronchiectasis diagnosis and chest HRCT within the past 5 years.	**Increase in cough**, **cough character change (dry to wet), or increased sputum** for ≥3 days, without **dyspnoea**, hypoxia (SpO2 <90%), or hospital admission.	No	No
ORBIT-3 and ORBIT-4 [7]<br><br>(Inhaled liposomal ciprofloxacin)	Adults with non-CF bronchiectasis, confirmed by chest CT.	Exacerbation Signs & Symptoms **(4+ for Abnormal)**: Four or more of the following: changes in **sputum**, **dyspnoea**, **cough**, fever (≥38°C), **wheezing**, **fatigue**, decreased FEV1/FVC, radiographic changes, or abnormal chest sounds. Mild= if there was no antibiotic use or macrolide dose increase. Moderate = treatment with antibiotics, or an increase in macrolide dose. Severe = treatment with intravenous antibiotics or hospital admission	Yes. In the case of a discrepancy between investigator assessment and protocol-defined criteria of a pulmonary exacerbation or its severity, adjudication was by a three-member committee blinded to the patient's group assignment.	No
BEST-1 [8]<br><br>(Amoxicillin-clavulanate versus azithromycin)	Children aged 1–18 years with CT-confirmed bronchiectasis	**Exacerbation** was defined as an increase in **cough frequency**, a change in character of the **cough** from dry to wet, or an increase in **sputum volume** or **purulence** for at least 3 days and unaccompanied by **dyspnoea**, hypoxia (SpO2 <90% in air), or need for hospital	No	No

		admission		
WILLOW [9]<br><br>(brensocatib)	Adults, with a clinical history consistent with bronchiectasis.	**EMBARC** definition.	No	Yes
ASPEN [10]<br><br>(Brensocatib)	Adults, BMI ≥18.5 kg·m−2, with a clinical history of bronchiectasis (cough, chronic sputum production, and/or recurrent respiratory infections), confirmed by CT scan radiologist examination.	**EMBARC** definition.	Yes. An independent clinical end point committee adjudicated all reported exacerbation events in a blinded manner to determine whether they met the protocol definition of an exacerbation and to determine the start and end dates for each event.	Yes
Foumani [11]<br><br>(Probiotics)	Adults with bronchiectasis identified by a pulmonologist	Not reported	Not reported	Not reported
AIRLEAF [12]<br><br>(CatC inhibitor)	Adults with CT-confirmed BE and history of pulmonary exacerbations requiring antibiotic treatment (either two or more exacerbations in the past year, or one exacerbation in the past year with a moderate-to-high symptom burden indicated by an SGRQ Symptoms score >40 at screening	**EMBARC** definition; time to first pulmonary exacerbation up to week 48	No	Yes
RESPIRE 1 [13]<br>deSoyza 2018	Adults with non-cystic fibrosis bronchiectasis, two or more	Exacerbations were required to meet three criteria: 1) worsening of at least three	No	no

(ciprofloxacin dry powder for inhalation)	exacerbations in the previous year and predefined bacteria in sputum	signs or symptoms (**dyspnoea**, wheezing, **cough**, 24-h sputum volume or sputum purulence) beyond normal day-to-day variation for at least 2 consecutive days, 2) fever (body temperature >38.0°C) or **malaise/fatigue** and 3) systemic antibiotic treatment ("primary end-point definition").		
RESPIRE 2 [14]<br><br>(ciprofloxacin dry powder for inhalation)	Adults with non-cystic fibrosis bronchiectasis, two or more exacerbations in the previous year and predefined bacteria in sputum	Exacerbations were required to meet three criteria: 1) worsening of at least three signs or symptoms (**dyspnoea**, wheezing, **cough**, 24-h sputum volume or sputum purulence) beyond normal day-to-day variation for at least 2 consecutive days, 2) fever (body temperature >38.0°C) or **malaise/fatigue** and 3) systemic antibiotic treatment ("primary end-point definition").	No	no
SAVE-BE [15]<br><br>(DPP-1 inhibitor HSK31858)	Participants were aged 18 years or older with a physician diagnosis of bronchiectasis, according to chest high-resolution CT showing bronchial dilatation and compatible respiratory symptoms, and at least two exacerbations within 12 months before screening.	**EMBARC:** annualised exacerbation frequency over 24 weeks	No	Yes
		**Ongoing trials**		
NCT03696290	Adults with CT confirmed BE	**EMBARC**	Not reported	Yes

(VitalBE)<br><br>(Inhaled aztreonam lysine)				
NCT07048262<br><br>(Nebulized CSL787)	Adults with CT confirmed BE	**EMBARC**	Not reported	Yes
NCT05407792<br><br>(Oral Staphylococcus Albicans)	Adults with CT confirmed BE	Not reported	Not reported	N/A
NCT07201051<br><br>(GSK3862995B)	Adults with CT confirmed BE	Not reported	Not reported	N/A
NCT06487273<br><br>(SIMEOX Device)	Adults with CT confirmed BE	Not reported	Not reported	N/A

Note: Some trials although published after the EMBARC criteria, had participant enrolment periods before the EMBARC criteria was published.
Note: Symptoms that are in bold text in column 3 are also part of EMABARC.

References for Supplement 1:

1. Haworth CS, Shteinberg M, Winthrop K, Barker A, Blasi F, Dimakou K, et al. Inhaled colistimethate sodium in patients with bronchiectasis and Pseudomonas aeruginosa infection: results of PROMIS-I and PROMIS-II, two randomised, double-blind, placebo-controlled phase 3 trials assessing safety and efficacy over 12 months. Lancet Respir Med. 2024 Oct;12(10):787-98. doi: 10.1016/S2213-2600(24)00225-X.
2. Liu Y, Lu HW, Gu SY, Wang WW, Ge J, Jie ZJ, et al. Bronchoscopic airway clearance therapy for acute exacerbations of bronchiectasis. EBioMedicine. 2021 Oct;72:103587. doi: 10.1016/j.ebiom.2021.103587.
3. Eklöf J, Alispahic IA, Sivapalan P, Wilcke T, Seersholm N, Armbruster K, et al. Targeted antibiotics for chronic pulmonary diseases (TARGET ABC): can targeted antibiotic therapy improve the prognosis of Pseudomonas aeruginosa-infected patients with chronic pulmonary obstructive disease, non-cystic fibrosis bronchiectasis, and asthma? A multicentre, randomized, controlled, open-label trial. Trials. 2022 Sep 27;23(1):817. doi: 10.1186/s13063-022-06720-z.
4. Eklöf J, Alispahic IA, Armbruster K, Lapperre TS, Browatzki A, Overgaard RH, et al. Systemic antibiotics for Pseudomonas aeruginosa infection in outpatients with non-hospitalised exacerbations of pre-existing lung diseases: a randomised clinical trial. Respir Res. 2024 Jun 6;25(1):236. doi: 10.1186/s12931-024-02860-9.
5. Nicolini A, Grecchi B, Banfi P. Effectiveness of two high-frequency chest wall oscillation techniques in patients with bronchiectasis: a randomized controlled preliminary study. Panminerva Med. 2022 Jun;64(2):235-43. doi: 10.23736/S0031-0808.20.03735-0.
6. Goyal V, Grimwood K, Byrnes CA, Morris PS, Masters IB, Ware RS, et al. Amoxicillin-clavulanate versus azithromycin for respiratory exacerbations in children with bronchiectasis (BEST-2): a multicentre, double-blind, non-inferiority, randomised controlled trial. Lancet. 2018 Oct 6;392(10154):1197-206. doi: 10.1016/S0140-6736(18)31723-9.
7. Haworth CS, Bilton D, Chalmers JD, Davis AM, Froehlich J, Gonda I, et al. Inhaled liposomal ciprofloxacin in patients with non-cystic fibrosis bronchiectasis and chronic lung infection with Pseudomonas aeruginosa (ORBIT-3 and ORBIT-4): two phase 3, randomised controlled trials. Lancet Respir Med. 2019 Mar;7(3):213-26. doi: 10.1016/S2213-2600(18)30427-2.
8. Goyal V, Grimwood K, Ware RS, Byrnes CA, Morris PS, Masters IB, et al. Efficacy of oral amoxicillin-clavulanate or azithromycin for non-severe respiratory exacerbations in children with bronchiectasis (BEST-1): a multicentre, three-arm, double-blind, randomised placebo-controlled trial. Lancet Respir Med. 2019 Sep;7(9):791-801. doi: 10.1016/S2213-2600(19)30254-1.
9. Chalmers JD, Haworth CS, Metersky ML, Loebinger MR, Blasi F, Sibila O, O'Donnell AE, Sullivan EJ, Mange KC, Fernandez C, Zou J. Phase 2 trial of the DPP-1 inhibitor brensocatib in bronchiectasis. New England Journal of Medicine. 2020 Nov 26;383(22):2127-37.
10. Chalmers JD, Burgel P-R, Daley CL, et al Phase 3 Trial of the DPP-1 Inhibitor Brensocatib in Bronchiectasis.NEJM 392(16):1569-1581, 2025 Apr 24
11. Foumani AA, Jafari A, Nejad AT, Jafarinejhad A, Ziyapour S, Keivanlou MH, Afzalipour M. Effects of Probiotics on Clinical Manifestations of Bronchiectasis: A Randomized, Triple Blinded, Placebo-Controlled Clinical Trial. Tanaffos. 2023 Feb;22(2):221
12. Chalmers JD, Shteinberg M, Mall MA, O'Donnell AE, Watz H, Gupta A, Frahm E, Eleftheraki A, Rauch J, Chotirmall SH, Armstrong AW, Eickholz P, Hasegawa N, Sauter W, McShane PJ. Cathepsin C (dipeptidyl peptidase 1) inhibition in adults with bronchiectasis: AIRLEAF, a phase II randomised, double-blind, placebo-controlled, dose-finding study. Eur Respir J. 2025 Jan 2;65(1):2401551. doi: 10.1183/13993003.01551-2024. PMID: 39255990; PMCID: PMC11694546.

13. De Soyza A, Aksamit T, Bandel T-J, et al. RESPIRE 1: a phase III placebo-controlled randomised trial of ciprofloxacin dry powder for inhalation in non-cystic fibrosis bronchiectasis. Eur Respir J 2018; 51: 1702052 [https://doi.org/10.1183/13993003.02052-2017].
14. RESPIRE 2: a phase III placebo-controlled randomised trial of ciprofloxacin dry powder for inhalation in non-cystic fibrosis bronchiectasis
15. Zhong NS, Qiu R, Cao J, Huang YM, Zhou H, Xu XX, Xu JF, Ye H, Yang ZR, Gao LY, Shen Y, Xiao ZK, Xie SG, Lin DJ, Zhao L, Xiong H, Zhang XM, Li FQ, Guan WJ, Chalmers JD; SAVE-BE trial investigators. Effects of the DPP-1 inhibitor HSK31858 in adults with bronchiectasis in China (SAVE-BE): a phase 2, multicentre, double-blind, randomised, placebo-controlled trial. Lancet Respir Med. 2025 May;13(5):414-424. doi: 10.1016/S2213-2600(25)00019-0. Epub 2025 Mar 25. PMID: 40154523
