# Supplementary material for: Adjudication of exacerbations in bronchiectasis using modified EMBARC criteria in the CLEAR Trial
Source: ERJ Open Res. 2026 Sep 28;12(5):00361-2026. doi: 10.1183/23120541.00361-2026 (PMC13617598; doi:10.1183/23120541.00361-2026)
Supplement: Supplementary file 3 [file 00361-2026.SUPPLEMENT3.pdf]

Supplement 3 Table 1 Results of Pulmonary exacerbation (PE) per category

Classification of Pulmonary exacerbation (PE)	Criteria for PE category	n/462 (%)PEs from 203 patients
**Ful EMBARC PE**	Deterioration in at least three symptoms<br>+<br>Duration of Symptoms are present for 48 hours or more<br>+<br>Antibiotics	239/462 (52%)
**Partial EMBARC PE**	Deterioration in at least three symptoms<br>OR<br>Duration of all the reported symptoms were present for 48 hours or more.<br>+<br>Antibiotics<br>(i.e. If antibiotics were prescribed and there were either 1 or 2 symptoms, AND these reported symptoms have been present for >48hrs this is a Partial exacerbation)	32/462 (7%)
**Non EMBARC PE**	Reported as an exacerbation and antibiotics were prescribed, but Full or Partial criteria not met	80/462
**Unreported/missed PEs**	PEs identified (i)when antibiotic recorded on the concomitant medications log and/or (ii) the patient reported taking antibiotics during a study visit but they had previously not contacted the site to inform them or enable categorising the exacerbation.	111/462
**No PE**	Information on trial database but no antibiotics were prescribed.	34
